# Impact of an enhanced screening program on the detection of non-AIDS neoplasias in patients with human immunodeficiency virus infection

**DOI:** 10.1186/s13063-021-05777-6

**Published:** 2021-11-27

**Authors:** M. Masiá, S. Padilla, G. Estañ, J. Portu, A. Silva, A. Rivero, A. González-Cordón, L. García-Fraile, O. Martínez, E. Bernal, C. Galera, V. Boix Martínez, J. Macias, M. Montero, D. García-Rosado, M. J. Vivancos-Gallego, J. Llenas-García, M. Torralba, J. A. García, V. Agulló, M. Fernández-González, F. Gutiérrez, E. Martínez, IMPAC-NEO Study Group, Antonia Alcaraz-García, Ana Caicedo, Alexy Inciarte, Alfredo Espinosa, Ana López-Lirola, Concha Amador, Ana Silva, Antonio Navarro, Ángela Camacho, Aurora Pérez, Carlos Galera, Melissa Carreres, Camila Piatti, David Vinuesa, David Dalmau, Dácil García-Rosado, Marcos Diez-Martinez, Daniel Podzamczer, Ester Saez, Esther Fagúndez-Reloba, Federico García, Juan Flores, Gema García, Javier García-Abellán, Lucía Guillén, Gema Navarro, Inma González-Cuello, Helena Albendín, Inmaculada Ruiz-Cáceres, Isabel Machuca, Ignacio Santos, Itziar Sobron, Juan Emilio Losa, Juan Luis Gómez-Sirvent, Jose Ramon Blanco, Juan Macías, Juan Carlos Gainzarai, Lorena Martínez, Josep Mallolas, María del Mar Alonso-Socas, Belén Martínez-López, Araceli Fernández, María Jehovana Hernández-Rodríguez, Miguel Angel Moran, Marta Navarro, Reyes Pascual, Francisco Pasquau, Pilar Callau, Joaquín Portilla, Catalina Robledano, Jhon Rojas, Ricardo Pelazas, Sandra Cuellar, Santos Del Campo, Sonia Calzado, Santiago Moreno, Sofía Scévola, Guillemo Telenti, Zuriñe Ortiz

**Affiliations:** 1 Hospital General Universitario de Elche and Universidad Miguel Hernández de Elche, Elche, Spain; 2 Hospital General Universitario de Elche, Elche, Spain; 3 Hospital Universitario Araba, Vitoria-Gasteiz, Spain; 4 Bellvitge University Hospital-IDIBELL, L’Hospitalet de Llobregat, Spain; 5 Hospital Universitario Reina Sofía de Córdoba, Instituto Maimónides de Investigación Biomédica de Córdoba (IMIBIC) and Universidad de Córdoba, Córdoba, Spain; 6 Hospital Clinic de Barcelona, Institut d’Investigacions Biomèdiques August Pi I Sunyer (IDIBAPS), Barcelona, Spain; 7 Hospital La Princesa, Madrid, Spain; 8 Hospital General Universitario Santa Lucía de Cartagena, Murcia, Spain; 9 Hospital General Universitario Reina Sofía de Murcia, Murcia, Spain; 10 Hospital Virgen de la Arrixaca, Murcia, Spain; 11 Hospital General Universitario de Alicante, Alicante, Spain; 12 Hospital Universitario de Valme, Seville, Spain; 13 Hospital La Fe, Valencia, Spain; 14 Hospital Universitario de Canarias, Santa Cruz de Tenerife, Spain; 15 Hospital Ramon y Cajal and Ramon y Cajal Health Research Institute (IRYCIS), Madrid, Spain; 16 Hospital Vega Baja, Alicante, Spain; 17 Hospital Universitario de Guadalajara, Guadalajara, Spain

**Keywords:** HIV infection, Neoplasms, Cancer, Non-AIDS-defining cancers, Screening, Early detection of cancer

## Abstract

**Background:**

The incidence of non-AIDS defining cancer (NADC) is higher in people living with HIV (PLWH) than in the general population, and it is already one of the leading causes of death in the HIV-infected population. It is estimated that the situation will be aggravated by the progressive aging of PLWH. Early diagnosis through intensive cancer screening may improve the ability for therapeutic interventions and could be critical in reducing mortality, but it might also increase expenditure and harms associated with adverse events. The aim of this study is to evaluate an enhanced screening program for early diagnosis of cancer in PLWH compared to standard practice. The specific objectives are (1) to compare the frequency of cancer diagnosed at an early stage, (2) to analyze safety of the enhanced program: adverse events and unnecessary interventions, (3) to analyze the cost-utility of the program, and (4) to estimate the overall and site-specific incidence of NADC in PLWH.

**Methods:**

We will conduct a multicenter, non-blinded, randomized, controlled trial, comparing two parallel arms: conventional vs enhanced screening. Data will be recorded in an electronic data collection notebook. Conventional intervention group will follow the standard of care screening in the participating centers, according to the European AIDS Clinical Society recommendations, and the enhanced intervention group will follow an expanded screening aimed to early detection of lung, liver, anal, cervical, breast, prostate, colorectal, and skin cancer. The trial will be conducted within the framework of the Spanish AIDS Research Network Cohort (CoRIS).

**Discussion:**

The trial will evaluate the efficacy, safety, and efficiency of an enhanced screening program for the early diagnosis of cancer in HIV patients compared to standard of care practice. The information provided will be relevant since there are currently no studies on expanded cancer screening strategies in patients with HIV, and available data estimating cost effectiveness or cost-utility of such as programs are scarce. An enhanced program for NADC screening in patients with HIV could lead to early diagnosis and improve the prognosis of these patients, with an acceptable rate of unnecessary interventions, but it is critical to demonstrate that the benefits clearly outweigh the harms, before the strategy could be implemented.

**Trial registration:**

ClinicalTrials.gov NCT04735445. Registered on 25 June 2019

## Administrative information

Note: the numbers in curly brackets in this protocol refer to SPIRIT checklist item numbers. The order of the items has been modified to group similar items (see http://www.equator-network.org/reporting-guidelines/spirit-2013-statement-defining-standard-protocol-items-for-clinical-trials/).

**Table:** 

Title {1}	IMPact of the enhAnced sCreening program on the detection of non-AIDS NEOplasias in patients with Human Immunodeficiency Virus infection. IMPAC-NEO.
**Trial registration {2a and 2b}.**	NCT04735445
**Protocol version {3}**	Date: 25th June 2019. Version identifier: 1.1.
**Funding {4}**	This study has received funding by the Spanish Instituto de Salud Carlos III (Madrid, Spain) (PI18/01861). Co-funded by European Regional Development Fund - "A way to make Europe".
**Author details {5a}**	^1^: Hospital General Universitario de Elche and Universidad Miguel Hernández de Elche ^2^: Hospital General Universitario de Elche ^3^: Hospital Universitario Araba ^4^: Bellvitge University Hospital-IDIBELL ^5^: Hospital Universitario Reina Sofía de Córdoba, Instituto Maimónides de Investigación Biomédica de Córdoba (IMIBIC) and Universidad de Córdoba ^6^: Hospital Clinic de Barcelona, Institut d’Investigacions Biomèdiques August Pi I Sunyer (IDIBAPS) ^7^: Hospital La Princesa ^8^: Hospital General Universitario Santa Lucía de Cartagena ^9^: Hospital General Universitario Reina Sofía de Murcia ^10^: Hospital Virgen de la Arrixaca ^11^: Hospital General Universitario de Alicante ^12^: Hospital Universitario de Valme ^13^: Hospital La Fe ^14^: Hospital Universitario de Canarias ^15^: Hospital Ramon y Cajal and Ramon y Cajal Health Research Institute (IRYCIS) ^16^: Hospital Vega Baja ^17^: Hospital Universitario de Guadalajara
**Name and contact information for the trial sponsor {5b}**	Red Española de Investigación en SIDA (RIS). Email: ueielx@gmail.com
**Role of sponsor {5c}**	The study sponsor and funders do not have a role in study design; collection, management, analysis, and interpretation of data; writing of the report; and the decision to submit the report for publication, including whether they will have ultimate authority over any of these activities.

## Introduction

### Background and rationale {6a}

Non-AIDS-defining cancer (NADC) is an important cause of morbidity and mortality in people living with the human immunodeficiency virus (PLWH), being currently one of the most frequent causes of death [1–5]. The incidence of cancer in PLWH is 2–3 times higher than in the general population [6–8]. A systematic review, analyzing data from more than 600,000 PLWH and 10,891 new cases of cancer, confirmed that the incidence of NADC has progressively increased since the introduction of combined antiretroviral therapy (ART), probably reflecting better viral-immune control and aging associated with increase in overall survival of patients living with the virus [9].

The most frequent types of NADC in PLWH are lung cancer, hepatocellular carcinoma, anal carcinoma and cervical carcinoma, although some studies have suggested that there could also be a higher incidence and/or severity of other malignant tumors, such as breast, prostate, colorectal, or skin cancer, including melanomas [10, 11]. In the era of ART, lung cancer has become the most frequent and deadliest cause of NADC in PLWH [12, 13], and greater lethality has been documented in PLWH than in the general population [14]. It is estimated that at least 1 in 3 PLWH will die due to malignant neoplasms in the coming years [15, 16]. The causes of this increased incidence of NADC are not well known and there are several factors that could play a role, including immunosuppression, chronic inflammation and immune activation, ART exposure, higher rates of coinfection with oncogenic viruses, and traditional cancer risk factors such as smoking [17, 18].

Despite the progressive aging of PLWH and the increase in the incidence of cancer, there is currently no consensus on the optimal screening strategy of cancer in this population. Some of the clinical practice guidelines of PLWH, such as the Spanish Gesida [18] or the European AIDS Clinical Society [19], recommend the screening approaches that have shown benefit in the general population in terms of mortality or greater probability of therapeutic success. However, these benefits have not been confirmed in PLWH, in which these strategies could be insufficient. Moreover, in the general population, there are currently no established recommendations for the screening in two of the neoplasms of special concern in HIV-infected patients, i.e., lung and anal cancer. For this reason, it is necessary to generate scientific evidence that informs clinical practice guidelines on the best screening approach in PLWH.

We hypothesize that a specific enhanced screening strategy for NADC in PLWH could favor the early detection of malignancies, improving the ability for therapeutic interventions, and thus reduce morbidity and mortality for cancer in PLWH, with an acceptable rate of unnecessary investigations, and may be cost-effective. To address this hypothesis, we have designed a clinical trial, in which patients are randomized to one of two strategies: enhanced screening versus standard of care practice. This trial aims to determine if the benefits of the enhanced screening outweighs the harms and if it is cost-effective.

Relevant studies informing on the main NADC in PLWH and examining potential benefits and harms of screening interventions are described next. For lung cancer, a standardized incidence rate 2.5 times higher in PLWH than in the general population, after adjusting for smoking, has been reported [20]. Smoking remains the main risk factor associated with lung oncogenesis, also in PLWH. The National Lung Screening Trial (NLST), a randomized study in the general population with more than 53,000 participants, demonstrated a 20% reduction in lung cancer mortality and a 6.7% reduction in overall mortality in those undergoing lung cancer screening with 3 annual low-dose computed tomography (LDCT) compared to simple chest x-ray [21].

In Spain, 92% of cases of hepatocellular carcinoma (HCC) in PLWH occur in patients coinfected with hepatitis C virus (HCV) [22], and its incidence seems to be increasing [23]. It has been suggested that HCC could be more symptomatic and be diagnosed in more advanced stages in PLWH [24]. As screening strategy, semiannual ultrasound is recommended in patients with cirrhosis of any cause, including cases of sustained viral response (SVR) to HCV antiviral therapy but with established chronic liver disease [18], and in patients co-infected with hepatitis B virus (HBV) regardless of the stage of fibrosis [19].

The incidence of anal cancer in the group of men who have sex with men (MSM) reaches 144 cases/100,000 people-year, and reported risk factors, in addition to MSM, include infection with oncogenic human papillomavirus (HPV), smoking, and immunosuppression. The incidence of this cancer increased significantly with the availability of effective ART at the end of the 1990s and the increase in survival of PLWH and is much higher than in the HIV-negative population [25]. Preventive measures and screening programs in PLWH based on anal cytology and anoscopy are advocated by some experts [26] but evidence of benefit remains unknown.

For cervical cancer, traditional screening programs based on Papanicolaou smear or liquid based cervical cytology have demonstrated to reduce cervical cancer mortality and might be enhanced by HPV genotype testing [19]. HPV genotype may aid at determining the periodicity of cytology, so that if high-risk genotypes (HPV-HR) are detected, a semiannual cytology could be recommended. However, this strategy has neither been evaluated in PLWH.

Finally, although controversy remains on whether PLWH have a higher risk for breast, prostate, colon, and skin cancer, these neoplasms have been included in the trial.

### Hypothesis and objectives {7}

Hypothesis: an enhanced program for screening of NADC in PLWH can lead to early diagnosis of cancer and improve the prognosis, with an acceptable rate of unnecessary interventions and being cost-effective.

General objectives: to evaluate the efficacy, safety, and efficiency of an expanded screening program for early diagnosis of cancer in patients with HIV compared to usual practice, within the framework of the Spanish AIDS Research Network Cohort (CoRIS).

Specific objectives: (1) to compare the frequency of cancer diagnosed at an early stage of disease with extended screening versus usual practice, (2) to analyze safety of the program: adverse events and unnecessary interventions, (3) to analyze the cost-utility of the extended screening program, and (4) to estimate the overall and site-specific incidence of NADC in PLWH.

### Trial design {8}

This protocol has been designed as a multicenter, randomized, parallel-group, and superiority trial.

## Methods: participants, interventions, and outcomes

### Study setting {9}

The study will be carried in the framework of the Spanish network of AIDS research (CoRIS), including 30 centers, being all of them Spanish academic hospitals.

### Eligibility criteria {10}

Inclusion and exclusion criteria for participants:

Inclusion criteria: adult patients ≥ 18 years with confirmed HIV infection and who agree to participate in the study and sign the informed consent.

Exclusion criteria: any active AIDS-defining disease, current or past NADC, life expectancy < 5 years, pregnancy or lactation, patients who refuse to participate in the study, any other criteria that in the judgment of the clinician should prevent the patient to participate in the study.

Eligibility criteria for study centers: Spanish hospitals with HIV/AIDS Unit and Infectious Diseases/HIV (ID/HIV) specialists.

Eligibility criteria for study individuals who will perform the interventions: ID/HIV specialists, radiologists, gynecologists, and surgeons.

### Who will take informed consent? {26a}

Participants meeting the study criteria will be invited by their doctors in charge to join the study during their routine medical visits, regardless of time from HIV diagnosis and the standard screening procedures being carried out in their centers, explaining the possible benefits and risks of the trial, and they will obtain the informed consent to participate in the study.

### Additional consent provisions for collection and use of participant data and biological specimens {26b}

An additional consent will be provided for collection and use of participant biological specimens that will be stored in a biobank.

## Interventions

### Explanation for the choice of comparators {6b}

The trial will compare the frequency of NADC diagnosed at an early stage in patients undergoing an enhanced screening program compared with those following conventional standard of care screening according to the European AIDS Clinical Society recommendations.

### Intervention description {11a}

Conventional group:

a. Semi-annual ultrasound and alpha-fetoprotein for all patients with cirrhosis and those with HBV infection with any degree of fibrosis.

b. Digital rectal examination and anal cytology every 1–3 years for all MSM men and anoscopy in case of cytology abnormal.

c. Cervical cytology every 1–3 years to all sexually active women, at least in those between 25 and 64 years.

d. Mammogram every 1–3 years to women between 50 and 70 years.

e. Rectal digital exam ± prostate-specific antigen (PSA) every 1–3 years for all men> 50 years.

f. Fecal occult blood test every 1–3 years in people between 50 and 75 years.

Enhanced group:

a. Annual LDCT to patients over 40 years of age, active smokers or who have quit in the last 3 years, with an accumulated index ≥ 20 pack-years and without contraindications for thoracic surgery, and without lung infections in the last 2 months.

b. Semi-annual ultrasound and alpha-fetoprotein for all people with chronic liver disease with fibrosis ≥ F3 of any cause (including HCV infection) or with any fibrosis for patients with HBV. Serum will be collected from all participants to be able to subsequently perform additional determinations such as alpha-fetoprotein-L3, decarboxyproprombin, or alpha-L-fucosidase.

c. Semi-annual digital examination and anal cytology to all MSM men and anoscopy in case of abnormal cytology.

d. Semiannual cervical cytology to all sexually active women between the ages of 21 and 64, including cotest (cervical cytology and HPV test) from the age of 30 if available (when cotest is available, controls will be made annually).

e. Annual mammogram for women between 50 and 70 years.

f. Annual PSA and digital rectal exam to all men ≥ 50 years.

g. Annual fecal occult blood to people> 40 years.

h. Annual general inspection for skin lesions suggestive of malignancy.

### Criteria for discontinuing or modifying allocated interventions {11b}

Interventions will be discontinued in case of pregnancy or revocation of the informed consent.

### Strategies to improve adherence to interventions {11c}

Adherence to the appointments will be recorded in the medical history and a new appointment arranged for the missed visits. Mobile phone messaging reminders for attendance are recommended to the participating centers after missing two visits.

### Relevant concomitant care permitted or prohibited during the trial {11d}

All concomitant interventions needed for medical care during the trial are permitted.

### Provisions for post-trial care {30}

All the included patients in the study are PLWH under medical treatment. Then, after the study, they will be cared for by their providers. The Spanish National Health Service will provide the required medical care to the study participants should they suffer harm as a result of the participation in the trial.

### Outcomes {12}

Primary outcome: diagnosis with a new NADC at stage 1 or 2, overall and for the specific cancer sites: invasive malignancies of lung, anus, liver, cervix, breast, prostate, colorectal, ovary, and invasive melanomas of skin assessed through various exams, and compared as the proportion between intervention groups at 12, 24, and 36 months.

Secondary outcomes: (1) diagnosis with a new NADC at any stage or unknown stage, overall and for the specific cancer sites: invasive malignancies of lung, anus, liver, cervix, breast, prostate, colorectal, ovary, and invasive melanomas of skin assessed through various exams, and compared as the proportion between intervention groups at 12, 24, and 36 months; (2) diagnosis with new cases of cancer diagnosed at stage 1 and 2 related to all new cases of diagnosed cancer compared as the proportion between groups at 12, 24, and 36 months; (3) unnecessary invasive procedures (with non-diagnostic results) compared as the proportion in each treatment group at 12, 24, and 36 months; (4) adverse events and severe adverse events related to the study procedures compared as the rate in each treatment group at 12, 24, and 36 months, and (5) overall mortality and mortality from cancer at 12, 24, and 36 months.

### Participant timeline {13}

The participant timeline is presented in Table 1.

**Table 1:** Schedule of enrollment, assessments, and data collection for the period of the trial

	Study period							
Time points	Enrolment	Allocation	Post allocation (months)					
	-t_0_	t_0_	6	12	18	24	30	36
**Enrolment**								
Eligibility screen	X							
Informed consent	X							
Allocation		X						
**Assessments**								
**Conventional screening**								
Semi-annual ultrasound and alpha-fetoprotein for all people with cirrhosis hepatitis B virus infection with any degree of fibrosis				X		X		X
Digital rectal examination and anal cytology to all MSM every 1–3 years				X		X		X
Cervical cytology every 1–3 years to all sexually active women, at least 25 to 64 years old				X		X		X
Mammogram every 1–3 years to women between 50 and 70 years				X		X		X
Rectal digital exam ± PSA every 1–3 years for all men> 50 years				X		X		X
Fecal occult blood test every 1–3 years in people between 50 and 75 years				X		X		X
**Enhanced screening**								
Over 40 years of age, active smokers or who would have abandoned in the last 3 years, with an accumulated index ≥ 20 pack-years and without contraindications for thoracic surgery, and without recent lung infections in the last 2 months				X		X		X
Semi-annual ultrasound and alpha-fetoprotein for all people with chronic liver disease with fibrosis ≥ F3 of any cause (including HCV infection) or any fibrosis for hepatitis B virus			X	X	X	X	X	X
Digital examination and semi-annual anal cytology MSM			X	X	X	X	X	X
Semiannual cervical cytology to all sexually active women between the ages of 21 and 64, including cotest (cervical cytology and HPV test) from the age of 30 if available (in that case, controls will be made annually)			X	X	X	X	X	X
Annual mammogram for women between 50 and 70 years				X		X		X
PSA and annual digital rectal exam to all men ≥ 50 years				X		X		X
Annual fecal occult blood to people > 40 years				X		X		X
Annual general inspection for skin lesions suggestive of malignancy				X		X		X

### Sample size {14}

The incidence of NADCs in the previous studies SMART and ESPRIT in virologically suppressed patients with CD4> 350 and over 40 years old was approximately 0.04% during 5 years of follow-up [27]. An Italian study described an incidence of NADCs of 0.67 per 100 person-years of follow-up [28]. In the CoRIS cohort, with 31.228 person-years of follow-up, a total of 136 NADCs were diagnosed, and the incidence was 0.43 per 100 person-years of follow-up. In a study of lung cancer screening among smokers, the prevalence of lung cancer was 2.03% [29]. For the calculation of the sample size, we have estimated an incidence of cancer diagnosed at an early stage of 2% in in the intervention group and 1% in the control group. With a statistical power of 80% and a loss to follow-up percentage of 20%, it is estimated that for a unilateral contrast it would be necessary to include 2.319 subjects in the exposed group (enhanced intervention) and 2.319 in the unexposed group (standard intervention) (1:1), with a total sample of 4.638 patients.

### Recruitment {15}

This protocol corresponds to an independent clinical study carried out in the framework of the cohort of the Spanish AIDS research network. The main strategy to achieve an adequate number of patients to reach the calculate sample size is to disseminate the project among the centers participating in the cohort.

## Assignment of interventions: allocation

### Sequence generation {16a}

The study will be operated by an electronic data collection notebook (REDCap®). This software allows assigning random numbers to the recruited patients. Also, there is a plan restriction to ensure well-balanced proportions of patients and representation of genders per branch and center. Specifically, random allocation will be carried out in blocks of twenty patients with a 1:1 ratio to either intervention group in men and women .

### Concealment mechanism {16b}

The implementation of the allocation sequence is done by the REDCap® software. Also, there is a datasheet information available to be used as a guideline for the investigators.

### Implementation {16c}

The enrolment of the patients will be done by the involved physicians in the study. Each participant center has a research team to carry out the rest of the activities of the project and a central Contract Research Organization (CRO) gives support for all of them.

## Assignment of interventions: blinding

### Who will be blinded {17a}

This is an open label trial.

### Procedure for unblinding if needed {17b}

Unblinding is not needed.

## Data collection and management

### Plans for assessment and collection of outcomes {18a}

Assessment and collection of baseline, and other trial data, including outcomes measures, will be performed by the centers research teams using an electronic data collection notebook (REDCap®)

### Plans to promote participant retention and complete follow-up {18b}

Patients’ adherence to protocol will be closely checked during the CRO’s monitoring visits. The protocol includes a form with a list of outcome data to be collected for participants who discontinue or deviate from intervention protocols. To improve retention and minimize loss to follow-up, investigators are asked to contact patients when they do not attend the scheduled visits and provide them a new appointment.

### Data management {19}

CRO staff will train research teams on data entry and storage on the electronic database collection notebook (REDCap®) and promote data quality checks.

### Confidentiality {27}

The Health Authority, the Ethics Committee, and the medical monitors and/or auditors designated by the Promoter may access to data base in each center, in order to verify the accuracy and reliability of the data provided by the principal investigator about the subjects participating in the trial. The designated monitors and/or auditors will work according to the provisions of the Articles 39 and 40 of the Spanish Law (RD 1090/2015) and the Principal Investigator must ensure that monitors, auditors, or CROs respect the confidentiality rules of any information about the subjects of the study. Each center will facilitate access to this data to its Ethics Committee and to the inspectors of the competent health authorities.

### Plans for collection, laboratory evaluation, and storage of biological specimens for genetic or molecular analysis in this trial/future use {33}

The protocol includes a description of the study plans for collection, laboratory evaluation, and storage of biological specimens for laboratory analysis in the current trial and for future use in ancillary studies. The use of the samples in ancillary studies requires the sign of an additional informed consent. The collected samples will be: plasma for all patients and whole blood and peripheral blood mononuclear cell samples for ancillary studies. All the biological samples will be stored at − 80 °C. Most Spanish hospitals have their own biobank to storage the samples collected. All biobanks must fulfill the requirements of the Spanish Law for these facilities.

## Statistical methods

### Statistical methods for primary and secondary outcomes {20a}

The primary efficacy analysis will be the comparison of the proportion of cancers diagnosed at an early stage (1 and 2) in each group following the principle of intent to screen, defined as patients who accept to participate in the study and sign the informed consent, regardless of whether they are subsequently excluded due to loss of follow-up, non-adherence to study procedures, unwillingness to remain in the study, death, etc. A per protocol analysis including patients who complete the study will also be performed. A sample size of 2319 patients in each group has been calculated (expected incidence of early cancer 2% and 1% in enhanced and conventional groups, respectively; statistic power 80%; losses to follow-up 20%). Missing observations in the variables will be ignored in the analysis.

The secondary efficacy analysis will include the comparison of the incidence of cancer at any stage or unknown stage and the incidence of new cases of cancer diagnosed at stage 1 and 2 related to all new cases of cancer diagnosed.

The secondary safety analysis will compare the number of unnecessary invasive procedures, the rate of adverse events and severe adverse events, and overall mortality and mortality from cancer in both groups.

The secondary cost-utility analysis will be carried out following the recommendations of the guide for the economic evaluation of health technologies in Spain from the perspective of Spanish National Health System. The incremental cost-effectiveness ratio will be calculated, which is a measure of contrast between the increase in costs and the increase in effectiveness between the different screening alternatives compared. Statistical uncertainty will be expressed as the 95% confidence interval. Random samples of 100 patients from each group replaced 10,000 times will be projected on a graph to generate the increase in QALYs (quality-adjusted life year), the increase in costs, and the cost-effectiveness curve.

The analyses will be performed by a statistician blinded to patients’ allocation using the statistical packages SPSS® v.17 or higher, and R® 3.3 or higher. The statistical significance is set at *p* < 0.05.

### Interim analyses {21b}

Interim analyses for futility, safety, and efficacy are planned every 12 months. The analyses will be performed by an independent data monitoring committee-DMC (an HIV specialist, a statistician, and an oncologist). There are no prespecified rules for stopping the trial. The DMC will review efficacy and safety data at each interim analysis and report to the trial steering committee who will make the final decision to terminate the trial if there are sufficient safety concerns or futility.

### Methods for additional analyses (e.g., subgroup analyses) {20b}

Subgroup analyses will be performed for: age groups every 5 years, sex, status of smoking, risk group, CD4 range, CD4 nadir, and HIV viral load. Sensitivity analysis will also be performed on the uncertain values of the model, taking the most pessimistic and optimistic assumptions about long-term survival of each tumor. Sensitivity analyses related to internal validity will also be carried out in the following variables: quality of life after positive screening and after the diagnosis of NADCs and radiation-induced cancer deaths. To analyze the generalizability of the results, sensitivity analyzes will be carried out in the operative mortality and in various costs, such as the costs of screening, follow-up LDCT, surgery, chemotherapy, radiotherapy, and management of incidental findings. A sensitivity analysis will also be carried out using an annual discount of 0 and 5%. The sensitivity analyzes will be carried out through a probabilistic sensitivity analysis, and the results will be presented as a dispersion analysis (cost-effectiveness plan) and the acceptability curve.

The same statistical packages SPSS® v.17 or higher, and R® 3.3 or higher will be used to carry with these subgroup analysis. The statistical significance is also set at *p* < 0.05.

## Oversight and monitoring

### Composition of the coordinating center and trial steering committee {5d}

The steering committee, made up of eight academic investigators, developed the study protocol, will have full access to the interim data, is responsible for the decision to stop the trial, will publish the results, and wrote the manuscript. The committee members will vouch for the accuracy and completeness of the data reported. Endpoint adjudication will be performed by the investigators at the participating centers. The DMC may be asked to evaluate equivocal cases where the assignation of an outcome requires the judgment of an oncologist. Data management will be carried out by the CRO.

Steering Committee: Mar Masiá, Esteban Martínez, Sergio Padilla, Antonio Rivero, Onofre Martínez, Lucio García-Fraile, Enrique Bernal and Félix Gutiérrez.

### Composition of the data monitoring committee, its role and reporting structure {21a}

The DMC is composed of 3 members: an HIV specialist, a statistician, and an oncologist. The DMC will perform the interim analyses for futility, safety, and efficacy.

### Adverse event reporting and harms {22}

Possible adverse events and other unintended effects of the trial interventions will be reported by the investigators using the electronic database notebook of the study. In addition to unnecessary interventions, expected harms of the study include the potential complications associated with the invasive interventions derived from the abnormal findings observed in the diagnostic tests. Among the most frequent, pneumothorax or bleeding occurring after biopsy of a suspected malignant lung nodule found in the CT scan; bleeding after biopsy of a lesion found during anoscopy or colonoscopy (the latter, if indicated if positive fecal occult blood test), bacteremia/sepsis after prostate biopsy performed because of abnormal PSA values, etc. All harms occurring during the study will be reported, regardless of whether they were expected or not. Severe adverse events (SAE) need to be reported to the monitor of the study in a maximum of 24 h. The monitor will notify the SAEs to the coordinators of the study. The medical monitor will also be responsible to review the reported safety data, to obtain all the useful safety information not reported by the investigators and to advice the coordinators about violations of the protocol by any center and to document it. Harms will be reported in trial publications in the “Results” section of the manuscript

### Frequency and plans for auditing trial conduct {23}

Biannual reviews will be carried out by the CRO on all the data recorded by the researchers in the electronic database notebook.

### Plans for communicating important protocol amendments to relevant parties (e.g., trial participants, ethical committees) {25}

Important protocol amendments will be communicated to relevant parties: investigators, ethical committees, trial participants, and trial registries.

### Dissemination plans {31a}

Trial results will be communicated to healthcare professionals, participants, the public, and other relevant groups, via publication or other data sharing arrangements.

## Discussion

Related to general population, PLWH are at higher mortality risk due to screenable cancer [30]. Increased mortality can result from higher incidence or late diagnosis, the latter amenable to improved screening. However, the benefits and harms associated with cancer screening in PLWH are unclear and may differ compared with uninfected persons. This trial will help to determine whether the benefits of the enhanced screening outweigh the harms and if it is cost-effective for the Public Health Services.

Although the ideal outcome measure would be mortality due to cancer, with our sample size, we will not reach the number of patients needed to detect differences in mortality between both groups. Hence, we established as a main outcome measure the detection of a greater number of cancers in early stages. It is unlikely that a randomized controlled trial of screening will be conducted with enough power to determine the cancer mortality reduction among the HIV-infected population. This trial has other limitations. There could be differences in baseline NADCs screening in different centers. Attempts will be made to correct these differences by redefining the protocol of the control group using the baseline screening data of the baseline survey trying to unify the screening in that group. Additionally, as it is a controlled study with a parallel group, there may be a risk of benefit from the control group due to imitation of the intervention group. After the first interim data analysis and if strictly necessary, a historical control group can be included prior to the start of the intervention group.

## Trial status

The current protocol is the 1.1 version. The recruitment began on 25 June 2019, but unfortunately, it was disrupted by the COVID-19 pandemic state. Recruitment has been restarted in the different centers according to the COVID-19 pandemic situation on each area. Because of the overall delay in the recruitment, the duration of the study has been extended. The approximate date when the recruitment will be completed is by the end of December 2022. 
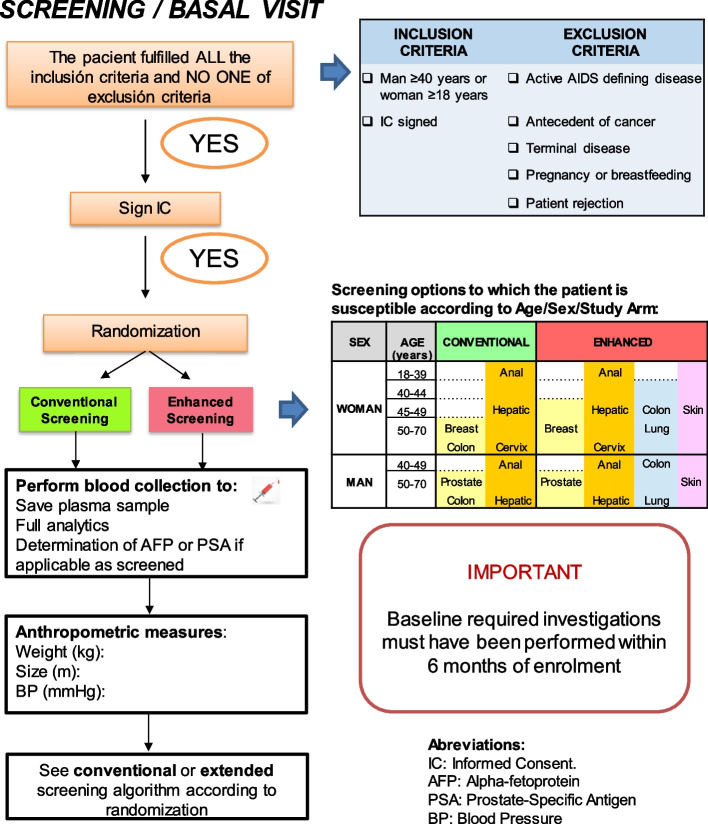


## Abbreviations

ART: Antiretroviral therapy
CoRIS: Cohort of the National AIDS Research Network
CRO: Clinical Research Organization
EACS: European AIDS Clinical Society
GP: General population
HBV: Hepatitis B virus
HCC: Hepatocellular carcinoma
HCV: Hepatitis C virus
HPV: Human papillomavirus
LDCT: Low-dose computed tomography
MSM: Men who have sex with men
NADCs: Non-AIDS-defining cancers
NHS: National Health Service
QALY: Quality-adjusted life year
PSA: Prostate-specific antigen
PLHIV: Population living with the human immunodeficiency virus
RIS: Spanish AIDS Research Network
