# Correction: Impact of an enhanced screening program on the detection of non-AIDS neoplasias in patients with human immunodeficiency virus infection

**DOI:** 10.1186/s13063-023-07655-9

**Published:** 2023-09-27

**Authors:** M. Masiá, S. Padilla, G. Estañ, J. Portu, A. Silva, A. Rivero, A. González-Cordón, L. García-Fraile, O. Martínez, E. Bernal, C. Galera, V. Boix Martínez, J. Macias, M. Montero, D. García-Rosado, M. J. Vivancos-Gallego, J. Llenas-García, M. Torralba, J. A. García, V. Agulló, M. Fernández-González, F. Gutiérrez, E. Martínez, IMPAC-NEO Study Group

**Affiliations:** 1 Hospital General Universitario de Elche and Universidad Miguel Hernández de Elche, Elche, Spain; 2 Hospital General Universitario de Elche, Elche, Spain; 3 Hospital Universitario Araba, Vitoria-Gasteiz, Spain; 4 Bellvitge University Hospital-IDIBELL, L’Hospitalet de Llobregat, Spain; 5 Hospital Universitario Reina Sofía de Córdoba, Instituto Maimónides de Investigación Biomédica de Córdoba (IMIBIC) and Universidad de Córdoba, Córdoba, Spain; 6 Hospital Clinic de Barcelona, Institut d’Investigacions Biomèdiques August Pi I Sunyer (IDIBAPS), Barcelona, Spain; 7 Hospital La Princesa, Madrid, Spain; 8 Hospital General Universitario Santa Lucía de Cartagena, Murcia, Spain; 9 Hospital General Universitario Reina Sofía de Murcia, Murcia, Spain; 10 Hospital Virgen de La Arrixaca, Murcia, Spain; 11 Hospital General Universitario de Alicante, Alicante, Spain; 12 Hospital Universitario de Valme, Seville, Spain; 13 Hospital La Fe, Valencia, Spain; 14 Hospital Universitario de Canarias, Santa Cruz de Tenerife, Spain; 15 Hospital Ramon y Cajal and Ramon y Cajal Health Research Institute (IRYCIS), Madrid, Spain; 16 Hospital Vega Baja, Alicante, Spain; 17 Hospital Universitario de Guadalajara, Guadalajara, Spain

**Correction: BMC Trials 22, 851 (2021)**

**https://doi.org/10.1186/s13063-021-05777-6**

Following publication of the original article [1], we have been informed that the correct name of Dr. Marta is Marta Fernández-González, not Marta González-Fernández.

Secondly, an additional source of funding was added in the "Funding" section of the Administrative information table.

The original article has been corrected.
